# Numerical Simulation of the 65Mn-Cr Steel Slab Solidification Process and Analysis of the Formation Mechanism of Internal Cracks

**DOI:** 10.3390/ma18040872

**Published:** 2025-02-17

**Authors:** Li Zhang, Lijun Xu, Guifang Zhang, Haibo Zhang, Qi Jiang, Shubiao Yin

**Affiliations:** 1 Faculty of Metallurgical and Energy Engineering, Kunming University of Science and Technology, Kunming 650093, China; zhangli2916@163.com (L.Z.); jiangqi87190500@163.com (Q.J.); yinshubiao@yahoo.com.cn (S.Y.); 2 National Engineering Research Center of Continuous Casting Technology, Central Iron and Steel Research Institute, Beijing 100081, China; 15731105523@163.com

**Keywords:** 65Mn-Cr steel, slab, flow–solidification coupling, equivalent plastic strain, intermediate crack

## Abstract

There are still internal defects such as triangular zone cracks, centerline cracks, and intermediate cracks in 65Mn-Cr steel during the production process, which mostly occur in the initial solidification. In order to explore the evolution of intermediate cracks during the initial solidification process of 230 mm × 1255 mm slab 65Mn-Cr steel, this study was based on a combination of numerical simulation and experiment, using COMSOL numerical simulation software to establish a flow and heat transfer coupling model and stress model, and carried out simulation research. The results show that the solidification speed of slab 65Mn-Cr steel is different at different positions from the meniscus. At the position where the reheating occurs, the heat transfer speed from the solidification front to the surface of the slab slows down, but the solidification speed varies in different areas of the section. At the same time, the flow field, temperature field, and cross-sectional stress and strain field are all non-uniformly distributed, and the maximum plastic strain value exceeds the critical strain 0.004. The experimental results show that internal cracks occur within the range of 9–35 mm below the surface. This shows that the intermediate crack defects of 65Mn-Cr steel are easily caused by stress and strain. Adjusting the spray distribution and cooling intensity of the spray water in the secondary cooling section can be a feasible solution to reduce the occurrence of internal cracks.

## 1. Introduction

With the development, acceleration of transformation, and upgrading of China’s manufacturing industry, the demand for fine-blanked parts has been continuously increasing. Especially in the automotive field, consumers’ requirements for improved vehicle quality have significantly increased the demand of automakers for quantity and quality of fine-blanked steel. 65Mn-Cr steel not only has high strength, toughness, and wear resistance, but it also has good elasticity, fatigue resistance, and workability. However, as a medium-high carbon steel, due to its relatively high carbon content, it is prone to internal defects such as triangular cracks, centerline cracks, and intermediate cracks [1]. These defects mostly occur in the initial solidification and secondary cooling zones, accounting for more than 50% of all types of defects. Moreover, most of the crack defects will expand during subsequent heating and rolling processes, ultimately leading to a decrease in the yield rate, which is a key problem restricting high-efficiency continuous casting production.

Cracks are generated under the combined action of internal and external factors. The internal cause is the reduction of the hot plasticity of the casting billet, including the formation of a liquid sulfide film at the solidification front [2], the aggregation of large-sized chain-like carbonitrides on the austenite grain boundaries [3], and the growth of a proeutectoid ferrite film on the austenite grain boundaries during the phase transformation process [4]. The external cause is mainly the result of the combined action of thermal stress, organizational stress, and mechanical stress during the solidification process of the molten steel [5,6,7]. The uneven temperature and inconsistent shrinkage between the surface and the interior of the continuous casting billet generate stress. When the tensile strain borne by the solidification front of the casting billet exceeds the critical value, or when the tensile stress exceeds the strength of the steel at the solidification temperature, cracks will form and expand [8]. Alternatively, a crack source is generated in the mold, and after passing through secondary cooling, the precipitation of low-melting substances causes the crack to form a secondary expansion. The repeated temperature rise of the surface layer of the casting billet will also cause multiple phase transformations, resulting in the formation and expansion of cracks along the interface of the two-phase structure.

However, some studies [9] have also shown that the internal cracks of the casting billet are directly related to the strength and plasticity of the solidification front. Experts such as Won [10], Seol [11], and Cornelissen [12] proposed a critical fracture stress model based on measured critical strain. This model takes into account the brittle temperature range and strain rate, and it analyzes the influence of the brittle temperature range and strain rate on the critical strain of internal cracks. Experts such as Yamanaka [13] and Kobayashi [14] believe that the sensitive area for the occurrence of casting billet cracks is the solidification front, and the solidification front is a temperature range, which is usually composed of the area between the zero ductility temperature (ZDT) and the zero strength temperature (ZST). Some scholars [15,16] believe that intermediate cracks are formed due to the well-developed columnar crystal structure of the casting billet, and the phase transformation from austenite to ferrite during the cooling process of the casting billet reduces the high-temperature plasticity of the steel, and cracks are formed under the action of external forces. Qin et al. [17,18] believed that the occurrence of intermediate cracks is related to various factors such as uneven cooling in the mold and the secondary cooling zone, the bulging of the continuous casting billet, and excessive straightening stress. Li et al. [19] summarized the experimental conclusions of their predecessors and believed that the occurrence of intermediate cracks is mainly due to the stress and strain caused by solidification shrinkage and that the critical stress range is 3.9–7.2 Mpa. Some scholars [20] established a thermo-viscoplastic finite element model, indicating that intermediate cracks are caused by the combined action of the tensile and compressive stresses of the billet shell and the expansion induced by the hydrostatic pressure of the molten steel and that the crack incidence can be reduced by optimizing the secondary cooling system. Niu [21] established a three-dimensional transient thermodynamic model, indicating that shrinkage makes the corner area of the wide face prone to longitudinal depressions. The occurrence of compressive plastic strain in the depression area and the large change in tensile plastic strain at the solidification front below the depression are the main reasons for the formation of intermediate cracks in the longitudinal over-corner depression.

At present, there are few reports on the simulation study of intermediate cracks during the initial solidification process of 65Mn-Cr steel slab. In this paper, a numerical simulation method is adopted. Aiming at the intermediate crack defects in the 65Mn-Cr steel slab during the production process, through establishing a flow, heat transfer, and thermomechanical coupling model, the evolution process of the intermediate cracks is simulated. The purpose is to explore the stress and strain behavior of the slab during the initial solidification process. The research results provide data support for optimizing process parameters on-site.

## 2. Mathematical Model

### 2.1. Model Assumptions

In this paper, a numerical simulation method is used to calculate the flow field and temperature field distribution of the casting blank during the continuous casting process by using COMSOL numerical simulation software of Version 6.1, and then the stress field is calculated. The causes of subsurface cracks in the casting blank are analyzed through the temperature field and stress field. Based on the 65Mn-Cr production process of a factory, a mathematical model is established for a continuous casting machine with a specification of 230 mm × 1255 mm slab.

According to the characteristics of the continuous casting process and the research purpose, the following assumptions are made for the flow and heat transfer calculations of the molten steel in the whole process:

(1) Ignore the radiation heat transfer between the surface of the molten steel and the surrounding environment and the heat transfer of the mold flux;
(2) Ignore the vibration of the mold and the influence of the taper;
(3) To simplify the calculation process, assume that the thermal conductivity of the solid-phase region of the casting blank is a function of temperature and the liquid-phase region is characterized by an equivalent thermal conductivity;
(4) Assume that the flow and heat transfer of the molten steel during the continuous casting process reach a stable state and the same cooling section is uniformly cooled.

Usually, when the casting blank exits the mold, the cooling method changes from mold copper wall cooling to spray cooling, and the cooling intensity changes greatly. The casting blank may be subjected to greater thermal stress. At the same time, due to the misalignment of the support rolls and the bulging of the casting blank, the mechanical stress increases, and the casting blank is extremely prone to cracks. Under the condition of ensuring the accuracy of the calculation results, the following simplified conditions for stress field solution are made based on the established three-dimensional temperature field assumptions of the slab:

(1) Assume that the internal medium of the casting blank is a continuous dense solid;
(2) The solidification shrinkage deformation behavior of the casting blank meets the requirements of the small deformation theory, and the yield follows the von Mises criterion. The incremental relationship between stress and strain of the casting blank under plastic yield follows the Prandtl–Reuss flow incremental theory under the plastic potential correlation;
(3) Ignore the influence of mechanical stress on the casting blank.

### 2.2. Control Equations

#### 2.2.1. Flow and Heat Transfer Model

The Continuity equation is as follows:

(1) $$\nabla \cdot \left(\rho u\right)=0$$

where *ρ* is the density, kg·m^−3^; *u* is the fluid velocity, m·s^−1^.

The Navier–Stokes equation is as follows:

(2) $$\rho \left(u\cdot \nabla \right)u=\nabla \cdot \left[-pI+K\right]+F+\rho g$$

where *K* is a function of the effective viscosity coefficient and the velocity component, where

(3) $$\mu_{eff}=\mu_{0}+\mu_{T}$$

where $\mu_{0}$ is laminar viscosity, kg·m^−1^·s^−1^; $\mu_{T}$ is turbulent viscosity, kg·m^−1^·s^−1^.

The specific equations of turbulent kinetic energy can be referred to in reference [22].

The energy conservation equation is as follows:

(4) $$\rho C_{p}u\cdot \nabla T+\nabla \cdot q=Q+Q_{P}+Q_{vd}$$

*F* is the Darcy source term,

(5) $$F=\frac{(1-{f_{l})}^{2}}{{(f}_{l}^{3}+0.001)}A_{mush}(u-u_{p})$$

where *f_l_* is the liquid fraction; *A_mush_* is a constant in the solid–liquid two-phase region, which has a great influence on the flow–solidification coupling calculation and is generally set to 1 × 10^4^−1 × 10^8^; *u* is the flow velocity of the molten steel, m·s^−1^; *u_p_* is the casting speed, m·s^−1^.

#### 2.2.2. Stress Field Model

During the continuous casting process, the solidification of the slab should consider problems such as thermal expansion deformation and elastoplastic deformation. Therefore, it is necessary to calculate the changes in stress and strain during the thermomechanical coupling analysis. According to the thermal elastoplastic incremental theory, the total strain increment expression of the casting blank is as follows:

(6) $$\varepsilon =\varepsilon_{e}+\varepsilon_{p}+\varepsilon_{T}$$

where ε is the total strain increment; $\varepsilon_{e}$ is the elastic strain increment; $\varepsilon_{p}$ is the plastic strain increment; $\varepsilon_{T}$ is the thermal strain increment.

In the elastic range, stress and strain should follow Hooke’s law, and the formula is

(7) $$\left\{\sigma \right\}=\left[D\right]\left\{\varepsilon \right\}$$

where $\left\{\sigma \right\}$ is the stress matrix; $\left[D\right]$ is the elastic coefficient matrix; $\left\{\varepsilon \right\}$ is the strain matrix.

In the plastic range, stress and strain satisfy the Prandtl–Reuss flow incremental theory under the von Mises criterion loading condition, and the mathematical expression is

(8) $$\varepsilon_{pl}=\lambda \frac{\partial \sigma_{e}}{\partial \sigma }$$

where λ is the plastic strain-related coefficient; $\sigma_{e}$ is the von Mises yield function.

The thermal elastic part is given by

(9) $$\varepsilon_{T}=\left[\alpha \right]∆T$$

where $\left[\alpha \right]$ is the thermal expansion coefficient matrix.

### 2.3. Simulation Process Parameters

The test steel grade is 65Mn-Cr, and its melting composition is shown in Table 1. The mold and process parameters are shown in Table 2.

### 2.4. Boundary Conditions

*V*_inlet_ is the normal inflow velocity at the inlet of the submerged nozzle, and it is determined by the casting speed and the cross-sectional size of the mold. Its calculation formula is

(10) $$V_{inlet}=V_{cast}\frac{S_{mold}}{S_{sen}}$$

The turbulent kinetic energy k and the turbulent kinetic energy dissipation rate ε at the inlet of the nozzle are calculated by the following formulas:

(11) $$k_{inlet}=0.01{V_{inlet}}^{2}$$

(12) $$\varepsilon =\frac{{{2k}_{inlet}}^{1.5}}{D}$$

where *D* is the diameter of the nozzle, m; *k* is the turbulent kinetic energy, m^2^·s^−2^; *S*_mold_ is the cross-sectional area of the mold, m^2^; *S*_sen_ is the cross-sectional area of the nozzle, m^2^; *V*_inlet_ is the inlet flow velocity of the nozzle, m·s^−1^; *ε* is the turbulent dissipation rate, m^2^·s^−3^.

The heat flux density of the mold is calculated according to the Savage [23] empirical formula:

(13) q=a−bhv_cast

The value of b is generally calculated according to the average heat flux density. According to the conservation of heat, the heat released by the mold is equal to the heat taken away by the cooling water of the mold. The average heat flux density $\bar{q}$ is calculated by the heat balance method:

(14) $$\bar{q}=\frac{Q_{w}C_{w}∆T_{w}\rho_{w}}{F}$$

(15) $$b=1.5(a-\bar{q})/\sqrt{t_{s}}$$

where *q* is the heat flux density of the mold, W·m^−2^; $\bar{q}$ is the average heat flux density of the mold, W·m^−2^; *Q*_w_ is the cooling water flow rate of the mold, m^3^·s^−1^; *C_w_* is the specific heat capacity of water, J·kg^−1^·K^−1^; Δ*T_w_* is the temperature difference between the inlet and outlet water of the mold, K; *ρ_w_* is the density of water, kg·m^−3^; *F* is the effective contact area between the water-cooled copper wall in the mold and the molten steel, m^2^.

Secondary cooling zone:

Water cooling zone:

(16) $$h_{w}=392.5W^{0.55}(1-0.0075T_{w})$$

Water mist zone:

(17) $$h_{w}=350W+130$$

In the above formulas, the unit of *h*_w_ is W·m^2^·°C; *W* is the cooling water flow rate, L·m^2^·s; *T*_w_ is the spray water temperature, °C; *T*_s_ is the surface temperature of the casting blank, °C;

Air cooling zone:

(18) $$q_{s}=\varepsilon \sigma \left[{\left(T_{b}+273.15\right)}^{4}-{\left(T_{0}+273.15\right)}^{4}\right]$$

where T_0_ is the ambient temperature, °C; ε is the emissivity of the slab surface, taken as 0.8; σ is the Boltzmann constant.

### 2.5. Physical Properties of 65Mn-Cr Steel

**Figure 1:**
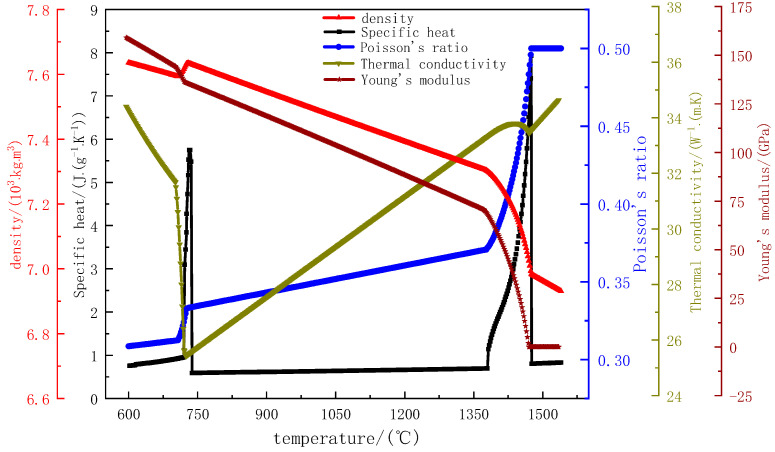
Physical property parameters of 65Mn-Cr steel.

**Figure 2:**
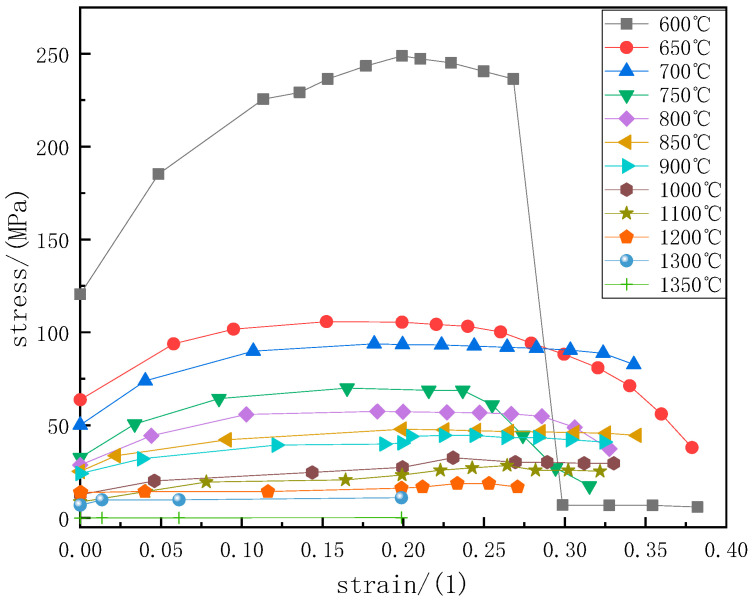
Stress–strain curve at high temperature of 65Mn-Cr.

The physical properties of 65Mn-Cr steel were calculated using JMatPro thermodynamic software. The results of parameters such as the density, thermal conductivity, elastic modulus, Poisson’s ratio, thermal expansion coefficient, and specific heat are shown in Figure 1. In this study, the thermal physical properties of 65Mn-Cr steel in the range of 600–1550 °C were selected. The elastic modulus and Poisson’s ratio are the basic parameters for describing the mechanical deformation behavior of materials. The elastic modulus gradually decreases from the solid-phase region to the liquid-phase region, and its value is 0 in the liquid-phase region. Poisson’s ratio gradually increases with the increase of temperature, and its value is 0.5 in the liquid-phase region. The stress–strain curve under high temperature conditions was obtained through experiments [24], as shown in Figure 2. In the range of 600–1000 °C, at the same temperature, the plastic stress rapidly increases with the increase of strain. When the strain exceeds 0.25, the stress reaches the tensile strength at that temperature. When the strain continues to increase, the stress decreases instead. At the same time, due to the poor plastic deformation ability of the casting blank at 600 °C, it is prone to sudden fracture when subjected to force. Therefore, when the strain exceeds 0.27, the stress drops to 0 MPa. In the range of 1000–1350 °C, the changes in stress and strain at different temperatures are not significantly different, and at the same temperature, the stress slowly increases with the increase of strain.

### 2.6. Model Verification

**Figure 3:**
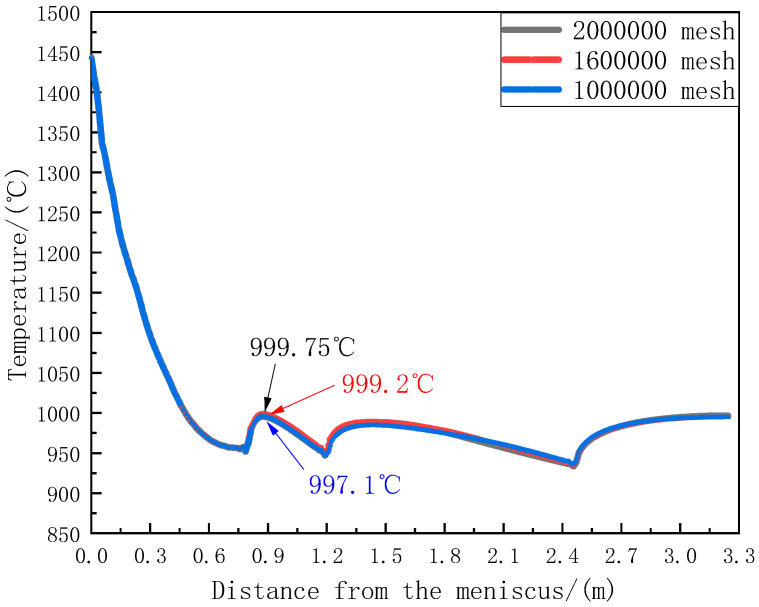
Verification of mesh independence.

To verify mesh independence, simulations were conducted using different mesh numbers, and the temperature results at the center of the wide face were compared. The comparison results are presented in Figure 3. At a position 0.9 m from the meniscus, when the mesh number is 1 million, the calculated temperature is 997.1 °C. When the mesh number increases to 1.6 million, the calculated temperature reaches 999.2 °C. When the mesh number is further increased to 2 million, the temperature reaches 999.75 °C. With a further increase in the mesh number, the temperature change is only 0.55 °C. Considering the computational speed of the workstation, and under the condition of ensuring relatively accurate model calculation results, 1.6 million meshes were selected for subsequent simulations.

**Figure 4:**
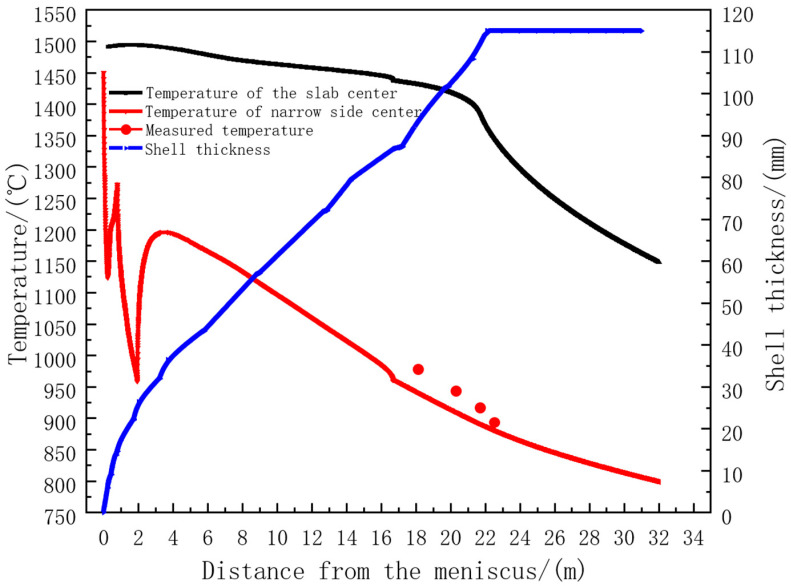
Comparison of calculated and measured temperature in the center of slab narrow surface.

The temperature change of the narrow face of the casting blank and the on-site temperature measurement results are shown in Figure 4. An on-site infrared thermometer was used to measure the temperature at the end of the 7th, 8th, and 9th sectors of the casting machine. The on-site measured average values were 978 °C, 943.3 °C, 916.6 °C, and 893.4 °C, respectively. The errors between them and the model calculation temperature values did not exceed 5%. The calculation errors were only 3.2% and 1.99%. At the end of the 9th sector, the model calculation temperature was 884.7 °C, and the error was only 0.97%. The specific data are shown in Table 3. Since the errors between the model calculation temperature and the on-site measured temperature from the end of the 7th sector to the end of the 9th sector were all within 5%, the calculation results of the model were relatively reliable.

## 3. Results and Discussion

### 3.1. Results of Flow–Heat Transfer–Solidification Coupling Calculation

**Figure 5:**
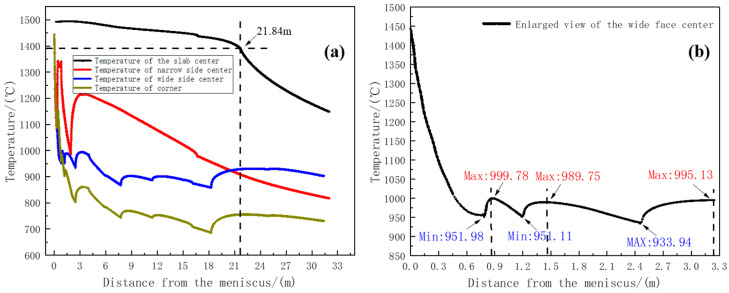
Temperature distribution curves: (**a**) temperature distribution curves at various positions of the slab; (**b**) enlarged temperature curve of the wide face.

The temperature change curves of four characteristic points of 65Mn-Cr continuous casting slab during the whole continuous casting process at a casting speed of 1.05 m/min and a specific water amount of 0.86 L/kg are shown in Figure 5. It can be seen from Figure 5 that when the distance from the meniscus is 21.82 m, the center temperature of the casting blank reaches the solidus temperature of 1380 °C, and the casting blank is completely solidified. Before the casting blank is completely solidified, the center temperature of the casting blank shows a slow downward trend. This is because the liquid core still has superheat in the mold and the first few zones of the secondary cooling section, and the heat is mainly released by the superheat. When the casting blank enters the sector section and the superheat disappears, the temperature is lower than the liquidus temperature, and the heat is mainly released by the latent heat. After the casting blank is completely solidified, the center temperature of the casting blank shows a significant downward trend. This is because the effects of superheat and latent heat are eliminated, and the casting blank rapidly releases heat. At the same time, due to the influence of the cooling of the wide and narrow faces of the spray water on the corner of the casting blank in the support roll section, the heat transfer speed is faster, so the temperature change is greater than that of the center of the wide face of the casting blank. When entering the second zone of the secondary cooling section, the corner is mainly affected by the heat transfer from the surface to the environment and the wide-face spray water, so the temperature change trend is the same as that of the center of the wide face.

According to the calculation results of the heat transfer model in Figure 5a, the temperature of the wide and narrow face centers shows a temperature return phenomenon in the first to fifth zones of the secondary cooling section. As shown in Table 4, the calculation data are the wide-face reheat temperature and reheat rate of each section of the casting blank. An enlarged view of the wide-face temperature distribution is shown in Figure 5b. A large temperature return occurs in the second zone of the secondary cooling section, and the reheat rate reaches 153.94 °C·m^−1^. The reheat rates in the first and third zones of the secondary cooling section decrease, being 118.05 °C·m^−1^ and 76.48 °C·m^−1^, respectively. Relevant studies have shown that the shell has a weak resistance to deformation at high temperatures. When the reheat temperature is too high, the greater the influence of the static pressure of the molten steel, the greater the change in the plastic strain at the solidification front. At the same time, in the temperature rise region, although the heat transfer rate from the solidification front to the surface of the casting blank slows down, there are differences in the solidification rates at different positions, resulting in differences in the shell thickness in different parts. The non-uniform shell thickness will generate thermal stress at the solidification front during the temperature return process. When this stress exceeds the critical stress that the casting blank can withstand, cracks will appear in the casting blank [25].

**Figure 6:**
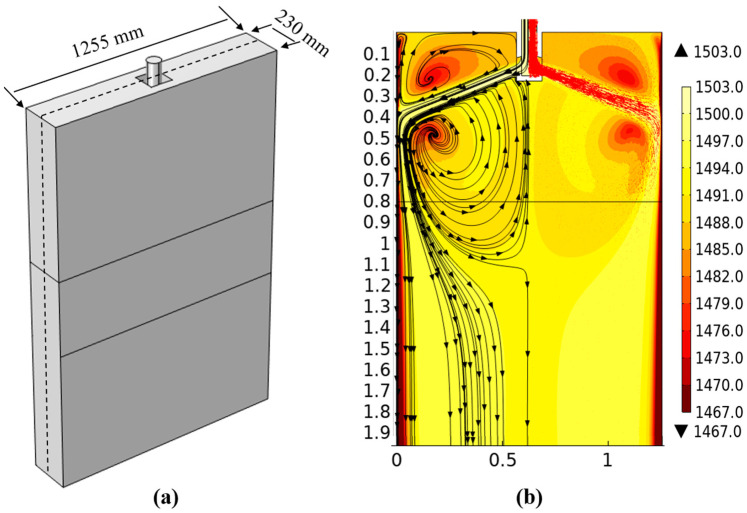
Flow field and temperature distribution at the central section of crystallizer: (**a**) geometric diagram; (**b**) flow field distribution and temperature distribution diagram.

Figure 6a is a geometric schematic diagram. Figure 6b is the temperature and flow field distribution nephogram of the central section of the mold. The molten steel flowing out from the submerged nozzle forms two upward and downward refluxes when washing the narrow side of the mold. At a position 0.37 m away from the meniscus, the solidified shell shows a reheating phenomenon due to the influence of the high-temperature jet molten steel, and the downward-flowing stream gradually separates at a position 1.1 m away from the meniscus at the lower part of the mold due to the continuous attenuation of its momentum. Part of the stream flows towards the lower end of the submerged nozzle, forming a large swirl zone. Because the temperature of the refluxing molten steel decreases, the temperature of the central region of the casting blank and the region near the narrow side is unevenly distributed. Another part of the stream flows deeper into the lower part of the mold and is evenly distributed on the entire cross-section of the casting blank [26,27].

**Figure 7:**
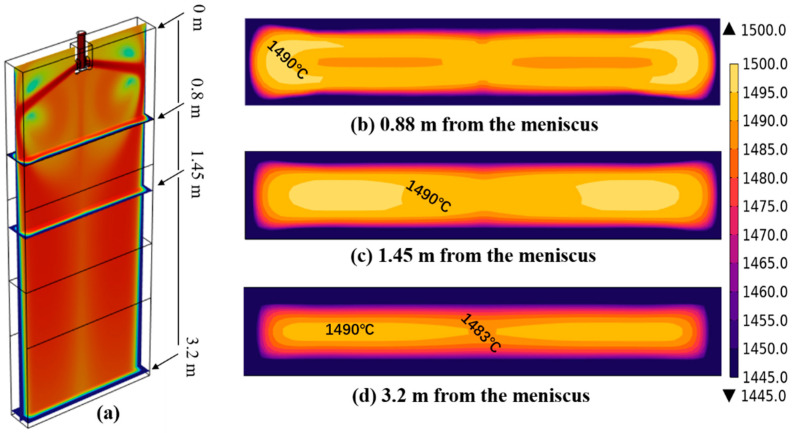
Temperature distribution of mold sections at different positions: (**a**) schematic drawings at different positions; (**b**) temperature profile at 0.88 m from the meniscus; (**c**) temperature profile at 1.45 m from the meniscus; (**d**) temperature profile at 3.2 m from the meniscus.

Figure 7a is a schematic diagram of the positions where the temperature return phenomenon occurs, which are 0.88 m, 1.45 m, and 3.2 m away from the meniscus, respectively. Figure 7b–d are the temperature distribution diagrams at different positions. Due to the jet action of the submerged nozzle, the molten steel still has a high temperature and a fast flow velocity, which causes the solidified shell generated in the mold to remelt. The temperature of the upward-refluxing molten steel decreases and the velocity slows down, having less influence on the shell at the center of the wide face. Therefore, the high-temperature region of the downward-flowing molten steel is larger than that of the center. At the same time, the temperature of the central region of the casting blank is lower than that of the downward-flowing molten steel stream. As shown in Figure 7b, the uneven temperature distribution in the cross-section of the casting blank can be observed at a position 0.88 m away from the meniscus. At a position 3.2 m away from the meniscus, with the increase of cooling intensity, the corner region is affected by the heat transfer in both the wide- and narrow-face directions, making the solidified shell of the cross-section approach a “square” shape. The position at 1/4 of the cross-section still remains in an overheated state, slightly higher than the temperature at the center position, showing a “dumbbell”-type distribution.

### 3.2. Stress–Strain Calculation Results During the Solidification Process

**Figure 8:**
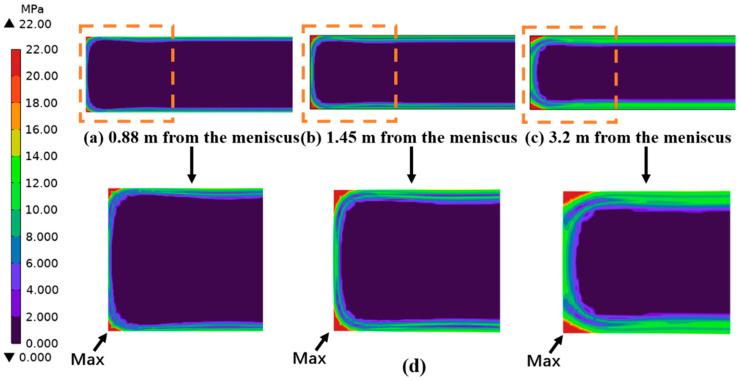
Stress distribution in different sections of the mold: (**a**) temperature distribution in sections 0.88 m away from the meniscus; (**b**) temperature profile at 1.45 m from the meniscus; (**c**) temperature profile at 3.2 m from the meniscus; (**d**) enlarged view of the narrow area.

As can be seen from the stress distribution in Figure 8, the cross-sectional stress distribution is in an uneven state affected by the flow–solidification interaction in the mold, and the stress change in the region near the narrow side is relatively large. The equivalent stress at the solidification front of the casting blank is mostly concentrated in the range of 2–6 MPa. The equivalent stress value in the corner region is much larger than that at the solidification front due to the two-dimensional heat transfer and lower temperature [28,29]. At the same time, as the solidification continues, when the distance from the meniscus is 3.2 m, the relatively large stress value in the corner region gradually extends to the center position of the wide face. The decrease of the surface temperature of the casting blank makes the stress value gradually increase.

**Figure 9:**
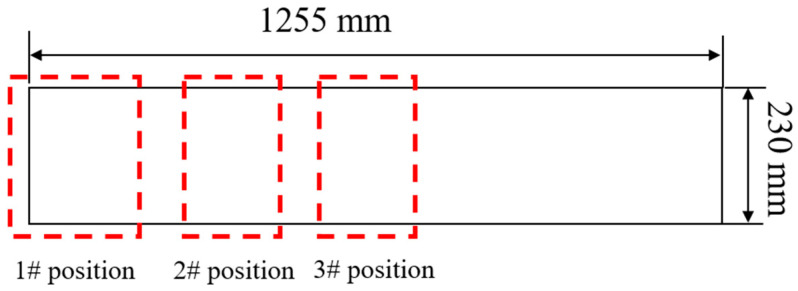
Location distribution of section 1#, 2#, and 3#.

To investigate the sensitivity of internal cracks in the slab at different positions, based on the critical strain theory and combined with the non-uniform distribution of the section temperature, the plastic strains in the range from ZDT (Zero Ductility Temperature) to ZST (Zero Strength Temperature) at the solidification front of the typical positions 1#, 2#, and 3# having relatively large section temperature non-uniformity were compared, and the crack sensitivity was analyzed simultaneously. The specific position distribution is shown in Figure 9.

**Figure 10:**
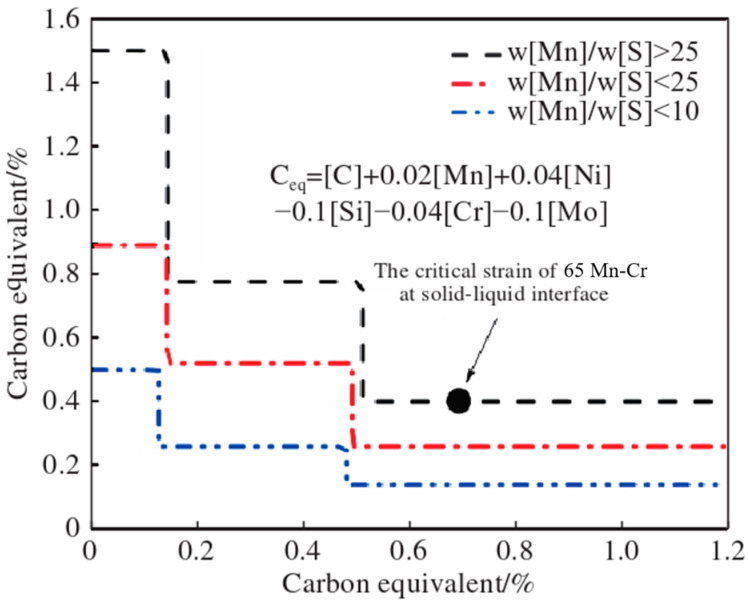
The relationship between carbon equivalent and critical strain.

In this study, 1, 2, and 3 represent the x, y, and z axes, respectively. S11, S22, and S33 represent the plastic strains perpendicular to the yz-plane, xz-plane, and xy-plane (along the width direction, thickness direction, and casting direction), respectively. The positive value is the plastic strain component under tensile stress, and the negative value is the plastic strain component under compressive stress [30]. Referring to the research of scholars [31] on the criteria for judging internal cracks, as shown in Figure 10, the critical strain value at the solidification front is obtained by calculating the carbon equivalent and manganese–sulfur ratio of the steel grade.

From the carbon equivalent formula in the figure,

(19) $$C_{eq}=\left[C\right]+0.02\left[Mn\right]+0.04\left[Ni\right]-0.1\left[Si\right]-0.04\left[Cr\right]-0.1[Mo]$$

The carbon equivalent of 65Mn-Cr can be calculated to be 0.644%. Since the manganese–sulfur ratio is greater than 25, the critical strain at its solidification front is calculated to be approximately 0.004. When the strain exceeds this critical value, crack defects are likely to occur inside the casting billet.

**Figure 11:**
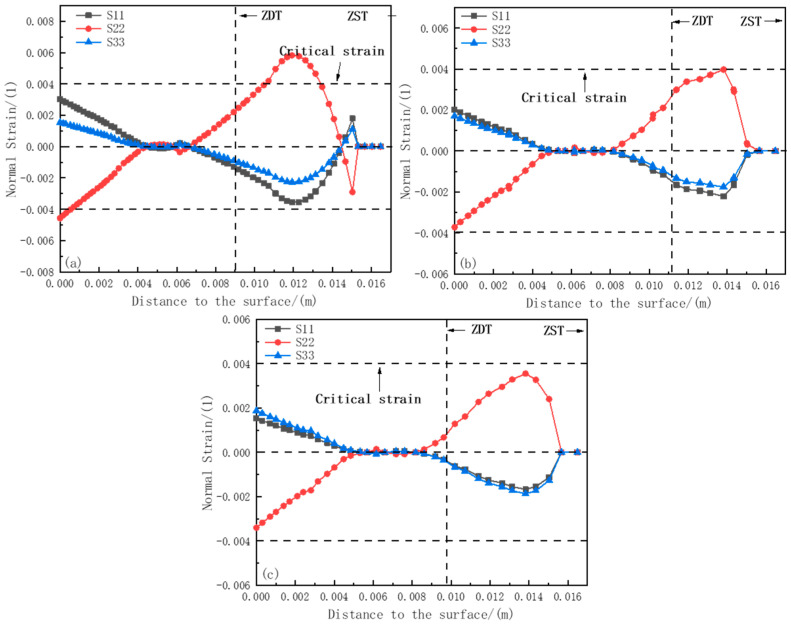
Strain component distribution at 0.88 m section: (**a**) strain distribution at section 1#; (**b**) strain distribution at section 2#; (**c**) strain distribution at section 3#.

Figure 11a–c show the strain component curves at positions 1#, 2#, and 3# of the 0.88 m section, respectively. At these positions, the phenomenon of plastic strain transformation occurs, indicating that the solidified shell is affected by both compressive stress and tensile stress. Meanwhile, at position 1# near the ZDT direction, the maximum value of S11 reaches 0.0058, exceeding the critical strain value of 0.004. While at positions 2# and 3#, the values of S22 are 0.00397 and 0.00355, respectively, which do not exceed the critical strain value. This means that at a position 0.88 m away from the meniscus, within the range from ZDT to ZST, position 1# is more prone to cracking, while positions 2# and 3# are less likely to produce cracks, and the relationship of the plastic strain components causing cracks is S22 > S11 > S33.

**Figure 12:**
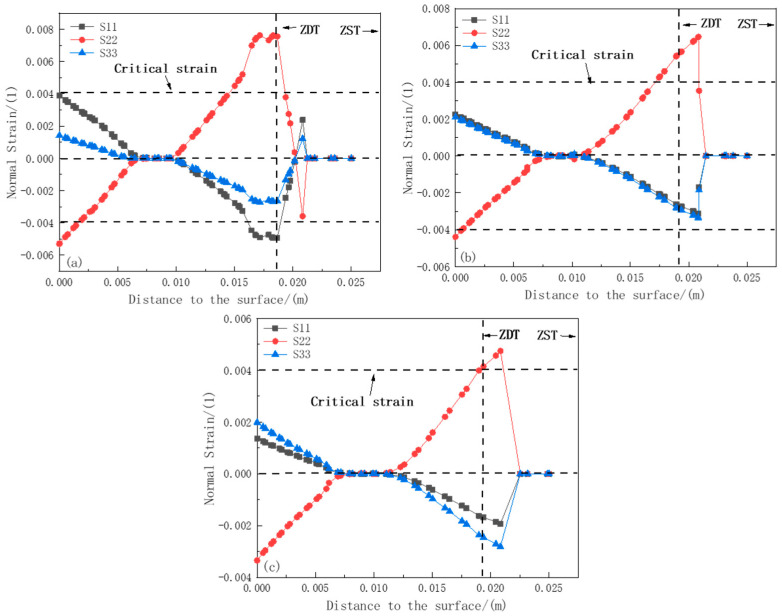
Strain component distribution at 1.45 m section: (**a**) strain distribution at section 1#; (**b**) strain distribution at section 2#; (**c**) strain distribution at section 3#.

Figure 12a–c show the strain component curves at positions 1#, 2#, and 3# of the 1.45 m section, respectively. Similar to the situation at the 0.88 m position, the phenomenon of plastic strain transformation occurs, which also indicates that the solidified shell is affected by both compressive stress and tensile stress. However, the plastic strain variation at position 1# is more intense. The maximum values of S11 and S22 near the ZDT direction at position 1# are 0.00763 and 0.0049, respectively, both exceeding the critical strain value of 0.004, while at positions 2# and 3#, the values of S22 reach 0.00647 and 0.0047, respectively, also exceeding the critical strain value. This indicates that at a position 1.45 m away from the meniscus, internal cracks are likely to occur within the range from ZDT to ZST, and the relationship of the plastic strain components causing internal cracks is S22 > S11 > S33. Cracks may appear along the thickness direction and the width direction inside the slab.

**Figure 13:**
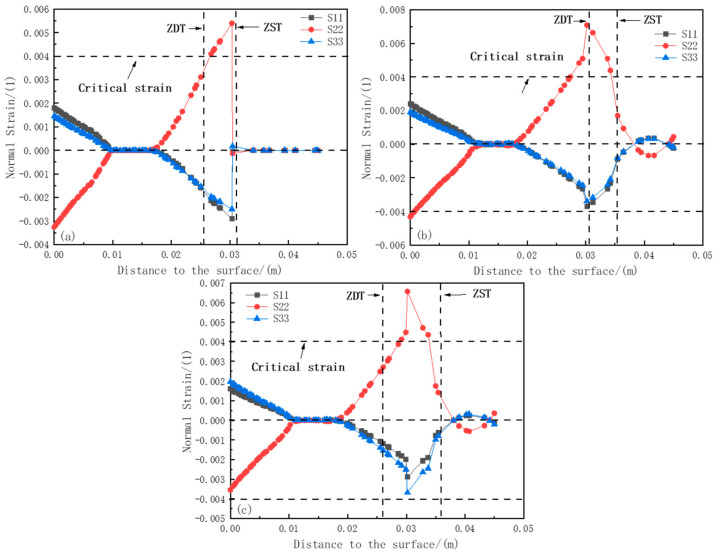
Strain component distribution at 3.2 m section: (**a**) strain distribution at section 1#; (**b**) strain distribution at section 2#; (**c**) strain distribution at section 3#.

Figure 13a–c show the strain component curves at positions 1#, 2#, and 3# of the 3.2 m section, respectively. Due to the decrease in temperature, the tensile and compressive resistance of the shell increases. The maximum value of S22 at position 1# within the range from ZDT to ZST is 0.00539, exceeding the critical strain value of 0.004. Meanwhile, the values of S22 at positions 2# and 3# reach 0.00708 and 0.00657, respectively, also exceeding the critical strain value. However, the values of S11 and S33 at these three positions do not exceed the critical strain value. This indicates that at a position 3.2 m away from the meniscus, internal cracks are likely to occur within the range from ZDT to ZST, and the relationship of the plastic strain components causing internal cracks is S22 > S11 > S33.

### 3.3. Experimental Results and Discussion

**Figure 14:**
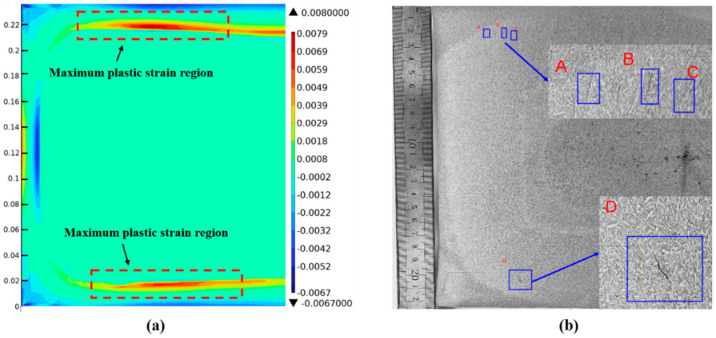
Position results of casting slab 1#: (**a**) strain distribution cloud; (**b**) low-power pickling results.

**Figure 15:**
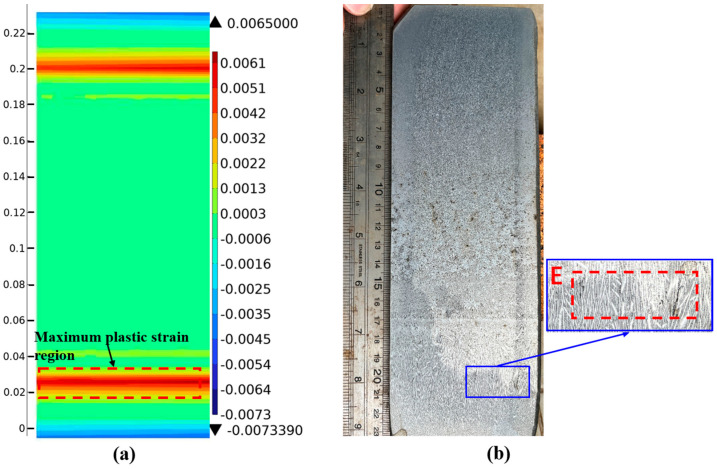
Position results of casting slab 2#: (**a**) strain distribution cloud; (**b**) low-power pickling results.

**Figure 16:**
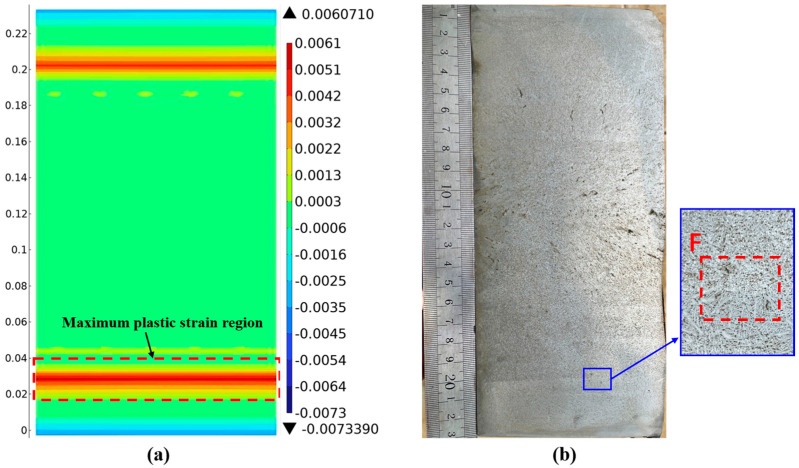
Position results of casting slab 3#: (**a**) strain distribution cloud; (**b**) low-power pickling results.

According to the inspection standards for macrostructure and pickling of casting slabs, an industrial hydrochloric acid aqueous solution with a volume ratio of 1:1 was prepared. When the temperature of the acid solution was in the range of 60–80 °C, macrostructure samples of the casting slabs at the typical positions 1#, 2#, and 3# having a size of 230 mm × 1255 mm were immersed in the acid solution for 30 min. Cracks were found at a depth of 30 mm below the surface of the casting slab, and the crack lengths were 10–20 mm, as shown in Figure 14, Figure 15 and Figure 16.

Figure 14a shows the strain distribution map at position 1# at 1.45 m. It can be seen that the maximum plastic strain region in the inner arc direction is about 15 mm away from the surface, that in the outer arc direction is about 20 mm away from the surface, and the maximum plastic strain is 0.008. Figure 14b shows the pickling results at position 1#. Subsurface cracks exist in both the inner arc and outer arc directions. Three fine cracks appear at 16 mm below the surface of the inner-arc wide face. The crack lengths at positions A and C are about 3 mm, and that at position B is about 4 mm. The directions of these cracks are all along the dendrite growth direction. A crack about 9 mm long appears at position D, 19 mm below the surface of the outer-arc wide face. Combined with the strain distribution map at position 1#, the plastic strain at the crack position is 0.0049, which exceeds the critical strain of 0.004. Subsurface crack defects appear in this region.

Figure 15a shows the strain distribution map at position 2# at 3.2 m. The maximum plastic strain region is about 31 mm away from the surface, and the maximum plastic strain is 0.0065. Figure 15b shows the pickling results at position 2#. A crack about 7 mm long appears at position E, 29 mm below the surface of the outer-arc wide face. Combined with the strain distribution map at position 2#, the plastic strain at the crack position is 0.0061, which exceeds the critical strain of 0.004. Subsurface crack defects appear in this region.

Figure 16a shows the strain distribution map at position 3# at 3.2 m. The maximum plastic strain region is about 31 mm away from the surface. In Figure 16b, two fine cracks about 3 mm long appear at position F, 33 mm below the surface of the outer-arc wide face. The two cracks are end-to-end and have a connection relationship, and the crack direction has no obvious relationship with the crystal growth direction. Combined with the strain distribution map at position 3#, the plastic strain at the crack position is 0.00607, which exceeds the critical strain of 0.004. Subsurface crack defects appear in this region.

According to the simulation results of the temperature field and stress–strain field during the solidification process of the casting slab, the thickness of the shell at the end of the region where the temperature rebounds from the mold to the middle position of the secondary cooling zone 3 is at most 32.3 mm. Combined with the fact that the subsurface cracks of the casting slab mainly occur in the range of 15–30 mm, it can be found that the crack positions are mainly concentrated in the first, second, and third sections of the secondary cooling zone. Based on the above analysis, the main reason for the occurrence of subsurface cracks in the casting slab is the non-uniform distribution of the steel liquid flow field inside the mold. At the same time, there are differences in the solidification rates between the corner region and the center of the wide face of the casting slab, resulting in differences in the shell thickness at different parts. The shell with non-uniform thickness will generate thermal stress during the subsequent solidification and contraction processes [32]. Therefore, on the premise of ensuring normal production, adjusting the spray distribution of the spray water in the secondary cooling zone can reduce the temperature difference at different positions of the casting slab, thereby reducing the occurrence of subsurface cracks.

## 4. Conclusions

(1) The solidification speeds of 65Mn-Cr steel slabs vary at different positions. Temperature rebounds occur at distances of 0.88 m, 1.45 m, and 3.2 m from the meniscus. Although the heat transfer rate from the solidification front to the surface of the slab slows down, differences in solidification speeds emerge at different positions. Moreover, due to the jet action of the submerged nozzle in the steel liquid, the flow field and temperature field distributions in the mold are non-uniform.
(2) The stress distribution across the section of the 65Mn-Cr steel slab is non-uniform. The stress at the solidification front of the slab is mostly concentrated in the range of 2–6 MPa. Meanwhile, at the typical positions 1#, 2#, and 3# having a certain degree of section temperature non-uniformity, the solidification front is mainly affected by the combined action of stresses in the width and thickness directions, and the plastic strain value exceeds the critical strain of 0.004, making the steel prone to the quality problem of internal cracks.
(3) The experimental results indicate that under the influence of the non-uniform distribution of the flow field and temperature field, crack defects appear within the shell thickness of 15–30 mm during the continuous casting production of 65Mn-Cr steel slabs. Therefore, on the premise of maintaining normal production, adjusting the spray distribution and cooling intensity of the spray water in the secondary cooling section to make it more coordinated with the flow field distribution of the steel liquid in the slab can effectively reduce the temperature difference at different positions of the slab, maintain the uniformity of the shell thickness, reduce the thermal stress at the solidification front, and decrease the occurrence of subcutaneous cracks.

## Figures and Tables

**Table 1:** Composition of 65Mn-Cr steel.

Element	C	Mn	S	P	Si	Cr	Ni	Cu	Mo
Content	0.64–0.68	0.95–1.1	0.01	0.02	0.2–0.37	0.13–0.22	0.3	0.25	0.1

**Table 2:** Mold and process parameters.

Parameter	Value
Casting blank section, mm × mm	230 × 1255
Casting speed, m·min^−1^	1.05
Submerged nozzle immersion depth, mm	120
Pouring temperature, °C	1503
Wide-face inner-arc water flow rate, L·min^−1^	3794.5
Wide-face outer-arc water flow rate, L·min^−1^	3785.1
Narrow-face northward water flow rate, L·min^−1^	495.59
Narrow-face southward water flow rate, L·min^−1^	496.53
Wide-face inner-arc temperature difference, °C	5.4
Wide-face outer-arc temperature difference, °C	5.1
Narrow-face northward temperature difference, °C	6.4
Narrow-face southward temperature difference, °C	6.2
Liquidus temperature, °C	1475
Solidus temperature, °C	1380

**Table 3:** Calculated temperature error values for each section.

Location	Calculated Temperature/°C	Measured Temperature/°C	Error
End of the 7th sector	944.7	978	3.4%
End of the 8th sector	912.8	943.3	3.2%
Middle of the 9th sector	898.4	916.6	1.99%
End of the 9th sector	884.7	893.4	0.97%

**Table 4:** Heat return and heat return rate of each stage.

Continuous Casting Machine Zone	Zone 1	Zone 2	Zone 3	Zone 4	Zone 5
Heat Rebound Temperature/°C	46.8	38.64	61.19	0.92	0
Heat Rebound Rate/(°C·m^−1^)	118.05	153.94	76.48	11.02	0

## Data Availability

The original contributions presented in this study are included in the article. Further inquiries can be directed to the corresponding authors.
